# Curettage and electrocoagulation *versus* surgical excision in the treatment of low-risk basal cell carcinoma – Postoperative follow-up and satisfaction at three months: randomized clinical trial^☆^

**DOI:** 10.1016/j.abd.2020.12.011

**Published:** 2022-02-28

**Authors:** Luan Moura Hortencio Bastos, Larissa Pierri Carvalho, Gabriela Roncada Haddad, Anna Carolina Miola, Juliano Vilaverde Schmitt

**Affiliations:** Department of Infectology, Dermatology, Diagnostic Imaging and Radiotherapy, Faculty of Medicine, Universidade Estadual Paulista, Botucatu, SP, Brazil

Dear Editor,

Several therapeutic approaches are currently described in the treatment of basal cell carcinoma (BCC), such as curettage and electrocoagulation (C&E), conventional surgery, Mohs micrographic surgery, topical or intralesional agents, radiotherapy, and photodynamic therapy. The choice of method depends on the BCC subtype, considering the size, location, clinicopathological pattern, and the patient clinical condition.^1^, ^2^

C&E has the advantages of shorter surgical time and easier approach to the lesion, requiring less facilities and material costs for it to be performed. However, the procedure has a longer healing or recovery time and may generate more unaesthetic scars when compared to conventional surgical procedures.^3^, ^4^

Although C&E has been classically described in the treatment of BCC, there are few studies prospectively comparing the technique with surgical excision. However, in observational studies, local recurrence rates for curettage and electrocoagulation have been shown to be comparable to those of conventional surgery for low-risk lesions.^2^, ^4^

Therefore, the present study performed a prospective, randomized, open, and controlled comparison between C&E interventions and conventional surgery for low-risk BCC in relation to surgical complications, postoperative recovery, aesthetic appearance, and patient satisfaction three months after the procedure.

Between February 2018 and March 2019, immunocompetent patients without coagulation disorders attended at the Outpatient Clinic of the Dermatology Service of Faculdade de Medicina de Botucatu ‒ UNESP with a diagnosis of low-risk, clinically evident BCC, measuring up to 10 mm in diameter were included in the study. The study was approved by the Research Ethics Committee of the institution (Counsel number: 4101709).

Low-risk lesions were those with well-defined edges, not a recurrence, outside of previously irradiated areas, located on the cervical region, trunk, extremities (except hands and feet), or low-risk regions on the face (forehead and cheek), with a pattern of nodular or superficial expansive growth.^2^

The lesions were outlined with a surgical pen, with a safety margin of 3 mm with the aid of a dermatoscope.

In the C&E group, the central portion of the lesion was excised with a tangential surgical blade incision (shaving) and sent for anatomopathological examination. The remainder of the tumor tissue was curetted until complete elimination on inspection, in addition to the surrounding skin up to a previous demarcation of three millimeters, or to the limit of friable tissue. Subsequently, the floor of the curetted area was electrocoagulated. The C&E process was repeated for a total of two cycles.^4^, ^5^

In the surgical excision group, the lesion was removed using a spindle-shaped incision, following the skin tension lines, guided by the demarcation of the three-mm safety margin, closed with direct sutures, using mononylon 4.0 (trunk and limbs) and 5.0 (face) using simple interrupted stitches.^6^

The patients were instructed to maintain local wound care by cleaning and dressing the wound with antibiotic ointment and to have the stitches removed, in the case of conventional surgery, at the basic health unit.

The studied characteristics were: sex; age; years of schooling; smoking status; phototype; clinical type of BCC; lesion diameter; signs of tumor recurrence; postoperative infection requiring local or systemic care; suture dehiscence; significant bleeding requiring health care; clinically evident scar retraction; pain intensity according to the analog scale from 1 to 10; pruritus according to the analogous scale from 1 to 10; the impact of postoperative care on the daily routine using a Likert-type question with the options almost never, a few times, many times, almost always; satisfaction with the surgical procedure using a Likert-type question with the options: none, a little, moderate, a lot and total; and aesthetic scar result at three months using the Patient and Observer Scar Assessment Scale and an analog scale, ranging from 0 to 10.^7^

The sample size calculation resulted in 98 lesions and was based on a difference in maximal satisfaction rate with the expected procedure of 60% *versus* 85% for a power test of 80% and p ≤ 0.05, two-tailed.

The characteristics with p < 0.2 in the bivariate analyses were analyzed by multivariate mixed models adjusted for age, sex, phototype, years of schooling, smoking status, size and location of lesions, with the intervention used as the dependent variable. Two-tailed values ​​of p ≤ 0.05 were considered significant.

A total of 116 lessons from 82 patients were included, 49% of which were female, with a mean of 1.4 lesions per patient (Table 1).

**Table 1:** Characteristics of the participants and lesions included in the study.

	C&E (n = 55)	Excision (n = 61)	RR (95% CI)	p
Female sex^a^	20 (37%)	29 (47%)	0.79 (0.51‒1.22)	0.30
Male sex	34 (63%)	33 (53%)	‒	–
Age (years)^b^	72 (61‒75)	71.5(62‒75)	‒	0.67
Phototype 1‒2^a^	13 (25%)	17 (28%)	0.86 (0.46‒1.61)	0.65
Phototype 3‒5	40 (75%)	43 (72%)	‒	‒
Years of schooling^b^	5 (3‒10)	4 (3‒11)	‒	0.43
Smoking status^a^	8 (15%)	7 (11%)	1.31 (0.50‒3.38)	0.57
Nodular clinical pattern^a^	40 (74%)	48 (77%)	0.95 (0.77‒1.17)	0.67
Superficial clinical pattern^c^	5 (9%)	3 (5%)	1.91 (0.47‒7.63)	0.47
Pigmented^b^	8 (23%)	7 (14%)	1.63 (0.65‒4.08)	0.29
Lesion diameter (mm)^b^	7 (5‒8)	7 (5‒9)		0.28
Head and neck^a^	31 (57%)	33 (53%)	1.07 (0.77‒1.49)	0.65
Trunk and UL	23 (43%)	29 (47%)	‒	‒

C&E, Curettage and Electrocoagulation.

a Chi-square test.

b Mann-Whitney test. Median (p25-p75%).

c Fisher exact test.

All lesions had the diagnosis of basal cell carcinoma confirmed on anatomopathological examination, and there were no clinical and dermoscopic signs of local recurrence or residual tumor at three months of follow-up.

Table 2 shows the characteristics of the lesions in terms of postoperative follow up, where there are no significant differences in the bivariate analyses. The characteristics with p < 0.2 were analyzed by multivariate mixed models adjusted for age, sex, phototype, years of schooling, smoking status, size, and location of the lesions. The variables pigmentation (p = 0.02), color change (p < 0.01), stiffness (p < 0.01), thickness (p = 0.02) and the patient total POSAS score (p = 0.01) were significantly associated with the surgical technique, showing worse scores for C&E.

**Table 2:** Characteristics assessed in the three-month postoperative period, according to the performed procedure.

	C&E (n = 55)	Excision (n = 61)	RR (95% CI)	p
Smoking^a^	8 (15%)	8 (13%)	1.14 (0.46‒2.85)	0.77
Infection^b^	0 (0%)	1 (1.6%)	‒	0.99
Dehiscence	‒	2 (3%)	‒	–
Significant bleeding	0 (0%)	0 (0%)	‒	–
Scar retraction^b^	2 (4%)	5 (8%)	0.45 (0.09‒2.27)	0.45
Recurrence or residual tumor^b^	0 (0%)	0 (0%)	–	–
PO pain (intensity)^c^	0 (0‒0)	0 (0‒0)	‒	0.34
Days of pain^c^	0 (0‒0)	0 (0‒0)	‒	0.45
PO pruritus (intensity)^c^	0 (0‒0)	0 (0‒1)	‒	0.77
Days of pruritus^c^	0 (0‒0)	0 (0‒2)	‒	0.82
Care – a few times^b^	1 (2%)	2 (3%)	0.57 (0.05 a 6.15)	0.99
Care – almost never	54 (98%)	59 (97%)	‒	‒
Satisfaction – total^b^	46 (85%)	53 (85%)	0.99 (0.85 a 1.15)	0.99
Satisfaction – very much	9 (15%)	8 (15%)	‒	‒
Aesthetic score^c^	10 (10‒10)	10 (10‒10)	‒	0.63
Scar (POSAS score)^d^				
Vascular^c^	2.5 (2‒4)	3 (2‒3)	‒	0.51
Pigmentation^c^	2 (1‒4)	2 (1‒2)	‒	0.17
Thickness	2 (2‒3)	2 (2‒3)	‒	0.78
Contour^c^	2 (1‒3)	2 (1‒3)	‒	0.81
Elasticity^c^	2 (1‒3)	2 (2‒3)	‒	0.61
Tenderness^c^	1 (1‒1)	1 (1‒1)	‒	0.54
Pruritus^c^	1 (1‒1)	1 (1‒1)	‒	0.74
Change of color^c^	3 (1‒5)	2 (1‒3)	‒	0.07
Stiffness^c^	2 (1‒3)	2 (1‒2)	‒	0.08
Thickness^c^	2 (1‒3)	2 (1‒2)	‒	0.11
Irregularity^c^	2 (1‒3)	2 (1‒3)	‒	0.46
Total observer^c^	11 (7‒17)	12 (8‒15)	‒	0.67
Total patient^c^	11 (8‒17)	10 (6‒14)	‒	0.13

C&E, Curettage and Electrocoagulation.

a Chi-square test.

b Fisher exact test.

c Mann-Whitney test.

d Patient and Observer Scar Assessment Scale.

Few studies have evaluated the degree of satisfaction or quality of life of patients with different treatments. A study carried out in 2007 evaluated the impact of different techniques on the patient quality of life through the 16-item Skindex one and two years after the procedure, finding worse results for C&E when compared to the conventional excision and Mohs surgery. It is important to emphasize that the lesions were not restricted in size or risk level, having a mean diameter of 9.9 mm.^8^

In the present study, the evaluation carried out three months after the procedure did not identify significant differences regarding the satisfaction with the treatment, as well as greater difficulties in local care, symptoms, or surgical complications, despite slight but significant differences regarding the scar aspect. It is believed that such observations may change over time, with the reassessment of these patients being relevant, as some scar aspects tend to improve over time, especially in the elderly.^9^, ^10^

The present study has limitations related to the single-center, non-blinded study design, and evaluation limited to three months. However, the results were controlled for clinical and demographic characteristics regarding satisfaction outcomes, complications, and scar aspect, indicating satisfactory results for C&E when compared to conventional surgery for the profile of the studied lesions.

## Financial support

None declared.

## Authors' contributions

Luan Moura Hortencio Bastos: Design of the study; collection of data; interpretation of data; drafting of the manuscript; approval of the final version of the manuscript.

Larissa Pierri Carvalho: Data collection; data interpretation; critical review of important intellectual content; approval of the final version of the manuscript.

Gabriela Roncada Haddad: Data collection; data interpretation; critical review of important intellectual content; approval of the final version of the manuscript.

Anna Carolina Miola: Data collection; data interpretation; critical review of important intellectual content; approval of the final version of the manuscript.

Juliano Vilaverde Schmitt: Design and planning of the study, data collection, or analysis and interpretation of data; critical review of important intellectual content, approval of the final version of the manuscript.

## Conflicts of interest

None declared.
